# Proposal of COVID-19 Clinical Risk Score for the management of suspected COVID-19 cases: a case control study

**DOI:** 10.1186/s12879-020-05604-4

**Published:** 2020-11-18

**Authors:** Sho Nakakubo, Masaru Suzuki, Keisuke Kamada, Yu Yamashita, Junichi Nakamura, Hiroshi Horii, Kazuki Sato, Munehiro Matsumoto, Yuki Abe, Kosuke Tsuji, Nobuhisa Ishiguro, Yasuyuki Nasuhara, Satoshi Konno

**Affiliations:** 1 Department of Respiratory Medicine, Faculty of Medicine and Graduate School of Medicine, Hokkaido University, North 15 West 7, Kita-ku, Sapporo, 060-8638 Japan; 2 Division of Infection Control, Hokkaido University Hospital, Sapporo, 060-8638 Japan; 3 Division of Hospital Safety Management, Hokkaido University Hospital, Sapporo, 060-8638 Japan

**Keywords:** COVID-19, Clinical score, CT imaging, White blood cell, Eosinophil, Procalcitonin, Polymerase chain reaction test

## Abstract

**Background:**

No clinical scoring system has yet been established to estimate the likelihood of coronavirus disease (COVID-19) and determine the suitability of diagnostic testing in suspected COVID-19 patients.

**Methods:**

This was a single-center, retrospective, observational study of patients with suspected COVID-19 and confirmed COVID-19. Patient background, clinical course, laboratory and computed tomography (CT) findings, and the presence of alternative diagnoses were evaluated. Clinical risk scores were developed based on clinical differences between patients with and without COVID-19.

**Results:**

Among 110 patients suspected of having COVID-19, 60.9% underwent polymerase chain reaction (PCR) testing based on the judgment of physicians. Two patients were found to have COVID-19. The clinical characteristics of 108 non-COVID-19 patients were compared with those of 23 confirmed COVID-19 patients. Patients with COVID-19 were more likely to have a history of high-risk exposures and an abnormal sense of taste and smell. The COVID-19 group had significantly higher rates of subnormal white blood cell counts, lower eosinophil counts, and lower procalcitonin levels than the non-COVID-19 group. When blood test results, CT findings, and the presence of alternative diagnoses were scored on an 11-point scale (i.e., “COVID-19 Clinical Risk Score”), the COVID-19 group scored significantly higher than the non-COVID-19 group, more than four points in the COVID-19 group. All non-COVID patients who did not undergo PCR had a score of 4 or less.

**Conclusions:**

The COVID-19 Clinical Risk Score may enable the risk classification of patients suspected of having COVID-19 and can help in decision-making in clinical practice, including appropriateness of diagnostic testing. Further studies and prospective validation with an increased sample size are required.

## Introduction

The novel coronavirus disease (COVID-19) has been spreading rapidly since the first case of infection was confirmed in December 2019 in Wuhan, China, and cases are now being reported worldwide [1]. The cumulative number of COVID-19 infections has reached 36 million, with more than 10 million deaths reported [2]. In Japan, the number of infected people has been increasing, and outbreaks, not only in cities but also in medical and nursing care facilities, have become a public health problem [3]. The spread of infection in healthcare facilities can lead to facility disruption and the disintegration of healthcare systems in the community. Therefore, clinicians are required to properly identify and manage patients with COVID-19.

COVID-19 often causes fever, upper respiratory tract symptoms, cough, malaise, olfactory, and gustatory dysfunction after 7–14 days of the incubation period [4–7]. However, the clinical course and symptoms are nonspecific, making it difficult to distinguish COVID-19 from common cold and other febrile diseases. Although several hematologic and biochemical changes associated with COVID-19 have been reported [8–10], there is no specific blood test for COVID-19. Inflammatory marker levels and lymphocyte counts have been investigated as predictors of severe COVID-19 [11, 12], but few have been validated as tools for the diagnosis of COVID-19. On the contrary, chest computed tomography (CT) has shown a high frequency of abnormalities, and several CT findings characteristic of COVID-19 have been reported [13–15]. However, there are several pathological conditions such as interstitial pneumonia and pulmonary edema that need to be differentiated.

The definitive diagnosis of COVID-19 was confirmed mainly by polymerase chain reaction (PCR) testing for severe acute respiratory syndrome coronavirus 2 (SARS-CoV-2) RNA. The sensitivity of the PCR test for COVID-19 is approximately 70% [16–18]; therefore, there is a risk of producing false-negative results. Thus, it is still challenging to manage suspected COVID-19 cases in the absence of a highly sensitive and specific diagnostic test system. In addition, limited medical and human resources make it difficult to perform PCR testing sufficiently in some facilities and geographical regions. To date, no useful clinical indicators have been established for the management of suspected COVID-19 cases in healthcare settings.

In response to the spread of COVID-19 infection in Hokkaido, Japan, Hokkaido University Hospital has systematically managed suspected COVID-19 cases under the leadership of respiratory physicians and infection control teams. In this study, we retrospectively analyzed the clinical characteristics and management details of suspected COVID-19 patients at Hokkaido University Hospital. We also compared the clinical, laboratory, and radiological characteristics of suspected and confirmed COVID-19 patients to clarify the differences. Based on these findings, we developed a clinical score (COVID-19 Clinical Risk Score) to ensure the accurate management of suspected COVID-19 patients.

## Methods

### Patients

This single-center, retrospective observational study was approved by Hokkaido University Hospital Division of Clinical Research Administration. The study included suspected or confirmed COVID-19 patients who had been treated between March 13 and May 31, 2020 at Hokkaido University Hospital in Sapporo, Japan. Suspected patients were those whose symptoms and CT findings raised concerns regarding the presence of COVID-19. SARS-CoV-2 RNA PCR was performed in cases of suspected COVID-19 when respiratory physicians (SN, KK, YY, JN, HH, KS, KT, MM) deemed it necessary based on a comprehensive assessment of clinical and imaging findings. Every decision was finalized after discussions with at least two respiratory physicians. A positive nasal swab or sputum specimen on PCR, performed at Hokkaido University Hospital or by referral, was required for the definitive diagnosis of COVID-19. We excluded COVID-19 patients who showed a significant level of recovery and those who had severe complications. Some of the PCR test data from the patients with COVID-19 included in this manuscript have been presented in our previous papers [19–21].

### Data collection

The demographic characteristics (age and sex) and clinical data (referral source, symptoms, days from onset, comorbidities, laboratory findings, and CT findings) were collected from medical records by investigators. Medical information of confirmed COVID-19 cases diagnosed outside our hospital was collected from the earliest available data after the onset of COVID-19 symptoms.

### CT imaging score

**Fig. 1:**
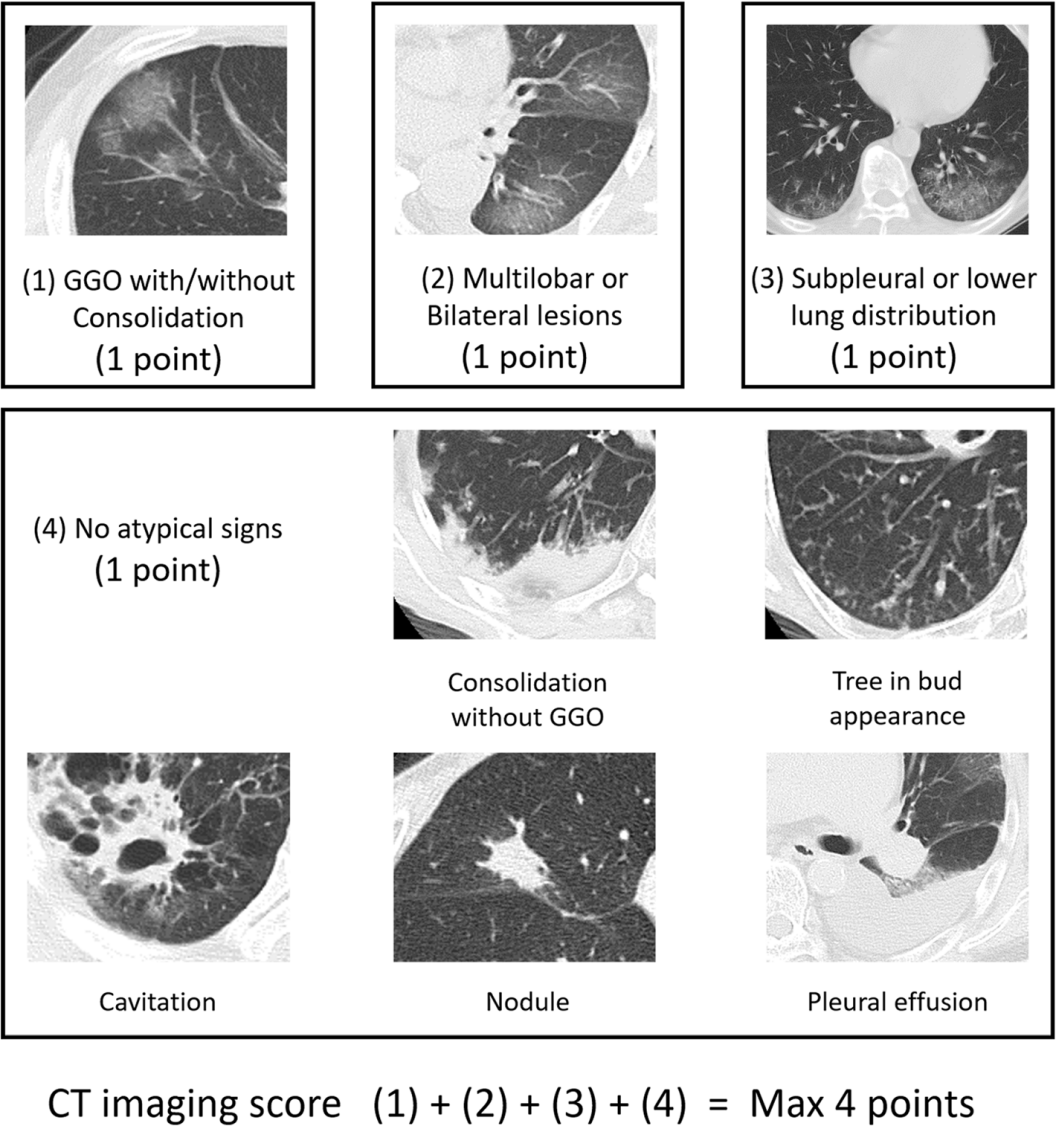
CT imaging score. All imaging findings were obtained from CT images of non-COVID-19 patients

Chest CT imaging findings that could be read by a non-specialist were selected and scored based on previous reports with modifications [13–15]. In addition to (1) ground-glass opacity (GGO) with or without consolidation, (2) multilobar or bilateral lesions, (3) subpleural or lower lung dominant distribution, and (4) absence of atypical findings (consolidation without GGO, hollow shadows, nodules, tree-in-bud appearance, or pleural effusion) were also taken into account. One point was added when each CT finding was observed, and the CT imaging score ranged from 0 to 4. Patients who had previous CT images were evaluated for newly appearing shadows. Each case was evaluated independently by two respiratory physicians to ensure consistency (Fig. 1).

### Statistical analysis

Descriptive statistics were determined for all study variables. All categorical variables were compared for study outcomes using the Fisher exact test, and continuous variables were compared using the *t* test or Mann-Whitney *U* test, as appropriate. Continuous data were expressed as the mean (standard deviation [SD]) or median (interquartile range [IQR]). Categorical data are expressed as proportions. A *P* value < 0.05 was considered statistically significant. JMP (SAS Institute Inc., Cary, NC, USA) and Prism 8 (GraphPad Software, San Diego, CA, USA) were used for statistical processing.

## Results

### Patient recruitment

**Fig. 2:**
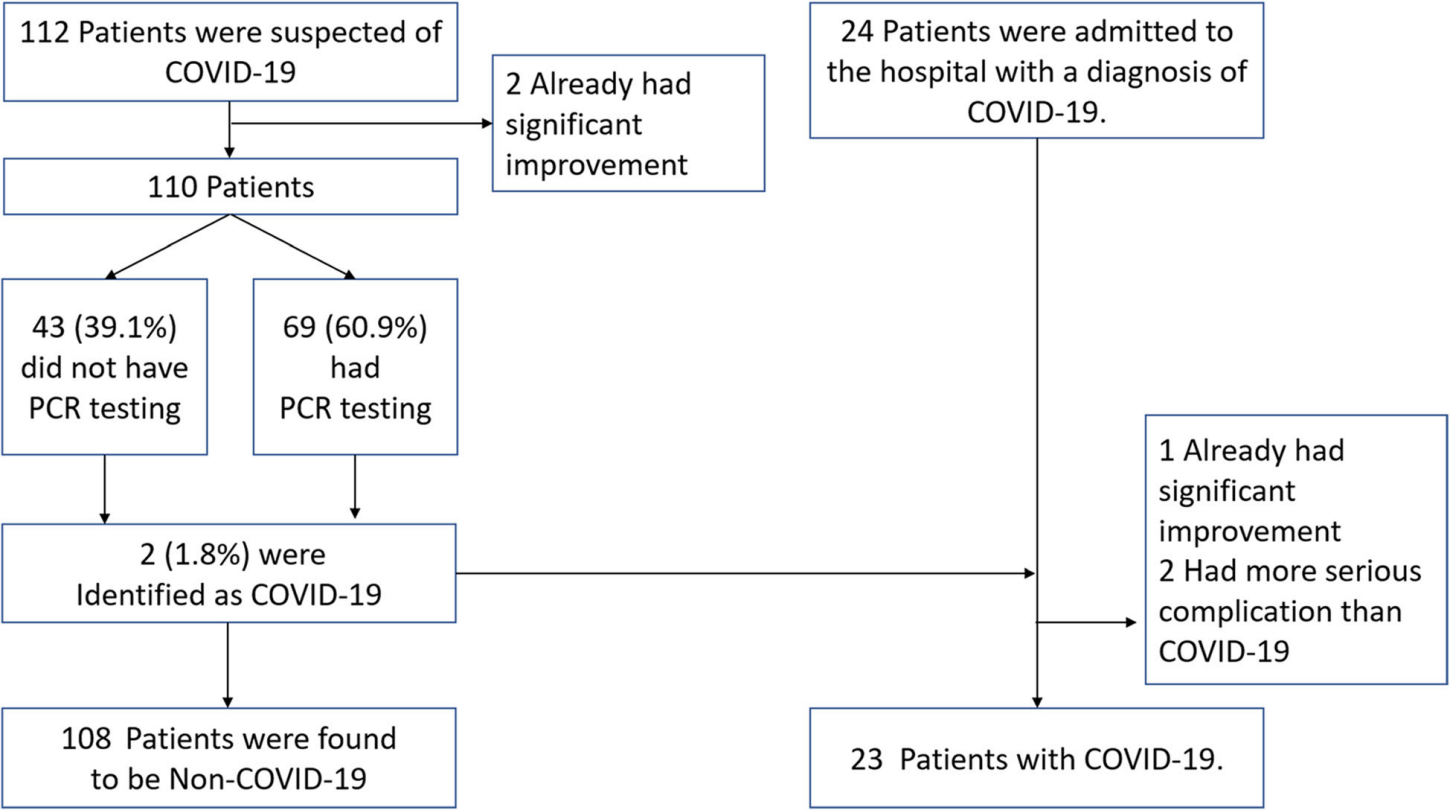
Flowchart of patient recruitment

Figure 2 shows a flowchart of patient recruitment. During the study period, 112 patients with suspected COVID-19 were referred to respiratory physicians; two of whom were excluded because their symptoms had already improved or resolved, so 110 patients were finally analyzed. Among the suspected cases, 60.9% underwent PCR testing based on the judgment of the respiratory physicians. Of these, only one patient showed a positive PCR result, and all others showed negative results. One patient with mild illness who was instructed to stay home without PCR was later found to have COVID-19 on PCR testing. Except for this patient, no other PCR-negative patients or those who did not undergo PCR were subsequently determined to have confirmed COVID-19.

### Analysis of cases

The median age of the suspected COVID-19 patients was 68.5 (range: 15–89 years), and 76.4% were male. The departments of referral were internal medicine (43.6%), surgery (30%), and emergency medicine (15.4%). Only 8.2% of the patients had a history of contact with COVID-19 patients, travel to endemic areas, or other high-risk behaviors. Most patients (88.2%) had underlying diseases, with malignancy (32.7%) being the most common. The most frequent presenting symptom was fever (72.4%), followed by cough (28.1%) and dyspnea (20.0%); 12.7% did not show any obvious symptoms. These asymptomatic patients were referred for abnormal chest CT findings. Among all patients, 26.3% had respiratory failure. These results, along with the results of the blood tests, are listed in Table 1. A chest CT was performed in all cases except one, and new abnormal findings were identified in 88.1% of the cases. The mean CT imaging score was 2.01. Based on the patient’s background, clinical course, laboratory findings, and CT findings, the respiratory physicians comprehensively considered whether the patient was likely to have COVID-19 or other diagnoses or whether the diagnosis was difficult to determine. A likely alternative diagnosis was considered in 79 (71.8%) of the cases. The differential diagnoses included drug-induced disease, radiation pneumonitis, acute exacerbation of interstitial pneumonia, pulmonary edema, bacterial pneumonia, sepsis, gravity-dependent atelectasis, etc.

**Table 1:** Clinical characteristics of patients with suspected COVID-19

	Total (*N* = 110)	PCR (*n* = 67)	No PCR (*n* = 43)	*P* value
**Characteristics**				
Age, years	68 (50–78)	69 (51–76)	67 (40–78)	0.78
Male	84 (76.4)	54 (80.6)	30 (69.8)	0.25
**Comorbidities**				
Hypertension	32 (29.1)	22 (32.8)	10 (23.3)	0.39
Diabetes	28 (25.4)	14 (20.9)	9 (20.9)	1.00
Malignancy	36 (32.7)	20 (29.9)	16 (37.2)	0.53
Chronic lung disease	27 (24.5)	20 (29.9)	8 (18.6)	0.26
Chronic heart failure	12 (10.9)	6 (9.0)	6 (14.0)	0.53
Coronary artery disease	16 (14.5)	10 (14.9)	6 (14.0)	1.00
Chronic renal disease	17 (15.4)	10 (14.9)	7 (16.3)	1.00
Cerebrovascular disease	14 (12.7)	10 (14.9)	4 (9.3)	0.56
Immunodeficiency	21 (19.1)	10 (14.9)	11 (25.6)	0.21
**History**				
Days from onset of symptoms	3 (2–7)	3 (2–7)	5 (2–11.5)	0.32
High-risk exposure history	9 (8.1)	9 (13.4)	0 (0.0)	0.01
**Symptoms**				
Fever	80 (72.4)	47 (70.2)	27 (62.3)	0.53
Cough	31 (28.1)	18 (26.9)	14 (32.6)	0.53
Nasal congestion	3 (2.7)	2 (3.0)	2 (4.7)	0.64
Sore throat	4 (3.6)	2 (3.0)	2 (4.7)	0.64
Dyspnea	22 (20)	17 (25.4)	5 (11.6)	0.09
Anosmia or ageusia	2 (1.82)	1 (1.5)	1 (2.3)	1.00
No symptoms	14 (12.7)	7 (10.5)	7 (16.3)	0.39
**Respiratory failure**	29 (26.3)	23 (34.3)	7 (16.3)	0.049
**Laboratory values**				
White blood cell count, cells/μL	6600 (5000–9900)	7600 (5400–10,800)	7100 (6000–10,200)	1.00
Neutrophil count, cells/μL	4524 (3089–7380)	4709 (3144–9378)	4981 (3678–7500)	0.81
Lymphocyte count, cells/μL	1046 (697–1597)	1089 (677–1587)	1288 (780–1719)	0.50
Eosinophil count, cells/μL	58 (0–165)	91 (21–179)	66 (0–196)	0.55
Platelet count, ×10^4^/μL	18.9 (14.5–25.9)	19.5 (14.5–24.2)	19.7 (16.5–28.3)	0.26
Lactate dehydrogenase, IU/L	215 (177–319)	213 (169–329)	200 (166–250)	0.14
C-reactive protein, mg/dL	4.10 (0.61–8.14)	4.30 (1.26–8.38)	1.36 (0.18–7.03)	0.06
Procalcitonin, ng/mL	0.08 (0.03–0.46)	0.18 (0.04–3.88)	0.04 (0.02–0.44)	0.10
**CT imaging Score**	2.01 ± 1.23	2.46 ± 1.15	1.28 ± 1.05	< 0.001
**Differential diagnosis**				
High likelihood of COVID-19	5 (4.6)	5 (7.5)	0 (0.0)	0.007
High likelihood of alternative diagnosis	79 (71.8)	42 (62.7)	37 (86.1)	

Data are shown as median (interquartile range), mean ± SD, or number (%)

We examined whether there was a clinical difference between suspected COVID-19 patients who did and did not undergo PCR testing. Compared with the PCR group, the non-PCR group demonstrated a significantly low percentage of high-risk exposure history (0.0% vs. 13.4%, *P* = 0.01) and respiratory failure (16.3% vs. 34.3%, *P* = 0.049), CT imaging score (1.28 vs. 2.46, *P* < 0.001), and had a high proportion of cases with an alternative diagnosis (86.1% vs. 62.7%, *P* = 0.009) (Table 1).

Next, the data of confirmed COVID-19 patients were analyzed. As shown in Fig. 2, we treated 26 confirmed COVID-19 cases, including those identified from the group of suspected cases. Patients whose symptoms had already resolved or whose main cause of symptoms was another co-existing condition were excluded; data from 23 patients were finally analyzed. The median age was 68 (range, 27–97) years, and 69.5% were male. Fever (92.3%) was the most common symptom, followed by cough (47.8%), while anosmia or ageusia was reported in only three patients (13.3%). At the time of data collection, patients with respiratory failure accounted for 39.1% of the study sample. Chest CT was performed in 16 patients (61.5%), and the mean CT imaging score was 3.50. Based on clinical information prior to definitive diagnosis, only one patient (4.5%) had a dominant differential diagnosis (Table 2).

**Table 2:** Clinical characteristics of COVID-19 and non-COVID-19 patients

	COVID-19 (*n* = 23)	Non-COVID-19 (*n* = 108)	*P* value
**Characteristics**			
Age, years	68 (54–78)	68.5 (49–78)	0.84
Male	17 (70.8)	82 (75.9)	0.72
**Comorbidities**			
Hypertension	8 (34.8)	32 (29.6)	0.63
Diabetes	4 (17.4)	23 (21.3)	0.78
Malignancy	6 (26.1)	36 (33.3)	0.63
Chronic lung disease	5 (26.1)	27 (25.0)	1.00
Chronic heart failure	4 (17.4)	12 (11.1)	0.48
Coronary artery disease	3 (13.0)	16 (14.8)	1.00
Chronic renal disease	2 (8.7)	17 (15.7)	0.52
Cerebrovascular disease	3 (13.0)	14 (13.0)	1.00
Immunodeficiency	2 (8.7)	20 (18.5)	0.36
**History**			
Days from onset of symptoms	5 (3.5–7)	3 (2–7)	0.56
High-risk exposure history	15 (65.2)	9 (8.3)	< 0.001
**Symptoms**^**a**^	(*n* = 23)	(*n* = 94)	
Fever	21 (92.3)	78 (83.0)	0.15
Cough	11 (47.8)	30 (31.9)	0.22
Nasal congestion	2 (8.7)	2 (2.1)	0.25
Sore throat	4 (17.3)	4 (4.3)	0.047
Dyspnea	5 (21.7)	22 (23.4)	1.00
Anosmia or ageusia	3 (13.0)	1 (1.1)	0.02
**Respiratory failure**	9 (39.1)	29 (30.9)	0.46
**Laboratory results (measured value)**			
White blood cell count, cells/μL	5180 (4150–6180)	7300 (5575–10,425)	< 0.001
Neutrophil count, cells/μL	3518 (2474–4429)	4800 (3586–8106)	0.007
Lymphocyte count, cells/μL	866 (690–1149)	1145 (723–1655)	0.13
Eosinophil count, cells/μL	0 (0–11)	83 (20–180)	< 0.001
Platelet count, ×10^4^ /μL	16.1 (13.8–20.5)	19.6 (16.1–26.6)	0.05
Lactate dehydrogenase, IU/L	270 (226–390)	210 (168–283)	0.02
C-reactive protein, mg/dL	5.31 (1.5–10.7)	3.72 (0.57–7.38)	0.12
Procalcitonin, ng/mL	0.065 (0.05–0.08)	0.12 (0.03–0.62)	0.33
**Laboratory results (cut off value)**			
White blood cell count < 8000 μL	21/23 (91.3)	59/108 (54.6)	< 0.001
Lymphocyte count < 1000 μL	14/22 (63.6)	56/92 (60.2)	0.81
Eosinophil count < 50/μL	21/22 (95.5)	33/93 (35.5)	< 0.001
Lactate dehydrogenase > 250 IU/L	13/20 (56.5)	37 /108 (34.3)	0.04
Procalcitonin < 0.5 ng/mL	12/12 (100)	33/48 (68.6)	0.03
Procalcitonin < 0.5 ng/mL and C-reactive protein ≥0.5 mg/dL	11/11 (100)	21/36 (58.3)	0.009
**CT imaging findings**			
CT imaging score	3.50 ± 0.86	2.00 ± 1.23	< 0.001
**Differential diagnosis**			
High likelihood of COVID-19	10 (43.5)	4 (3.7)	< 0.001
High likelihood of alternative diagnosis	1 (4.3)	79 (73.1)	

Data are shown as median (interquartile range), mean ± SD, or number (%)

^a^Data analysis was performed on excluded asymptomatic cases

We then compared the clinical findings between patients who did not have COVID-19 and confirmed COVID-19 patients (Table 2). Since all patients with COVID-19 were symptomatic, asymptomatic patients in the non-COVID-19 group were excluded for comparison of symptoms. The COVID-19 group had a higher frequency of sore throat and olfactory or taste disorders than the non-COVID-19 group (17.3% vs. 4.3%, *P* = 0.047, 13.0% vs. 1.1%, *P* = 0.02, respectively,). On blood tests, white blood cell (WBC) count was higher in the non-COVID-19 group than in the COVID-19 group (median, 5180 vs. 7300, *P* < 0.001), and large differences in the percentage of WBC counts, up to 8000/μL, existed between the two groups (91.3% vs. 54.6%, *P* < 0.001). On the other hand, there was no significant difference in the rate of lymphocytopenia (63.6% vs. 60.2%, *P* = 0.81) or absolute lymphocyte count (median, 866 vs. 1145, *P* = 0.13) between the two groups. The reduction in eosinophil count (< 50/ μL) was seen in many of the COVID-19 patients and was more frequent than that in the non-COVID-19 patient group (95.5% vs. 35.5%, *P* < 0.001). Analysis of biochemical test results revealed that the percentage of lactate dehydrogenase > 250 IU/L and procalcitonin < 0.5 ng/mL was significantly higher in patients with COVID-19 (56.5% vs. 34.4%, *P* = 0.04, 100% vs. 68.6%, *P* = 0.03, respectively). The difference in the percentage of low procalcitonin levels was pronounced when the analysis was limited to cases with C-reactive protein (CRP) ≥0.5 mg/dL (100% vs. 58.3%, *P* = 0.009). The mean CT imaging score was higher in the COVID-19 patient group than in the non-COVID-19 group (mean, 3.5 vs. 2.0, *P* < 0.001). Moreover, a significantly higher proportion of patients in the non-COVID-19 group than in the COVID-19 group had an alternative diagnosis (4.3% vs. 73.1%, *P* < 0.001).

### Developing COVID-19 Clinical Risk Score

**Fig. 3:**
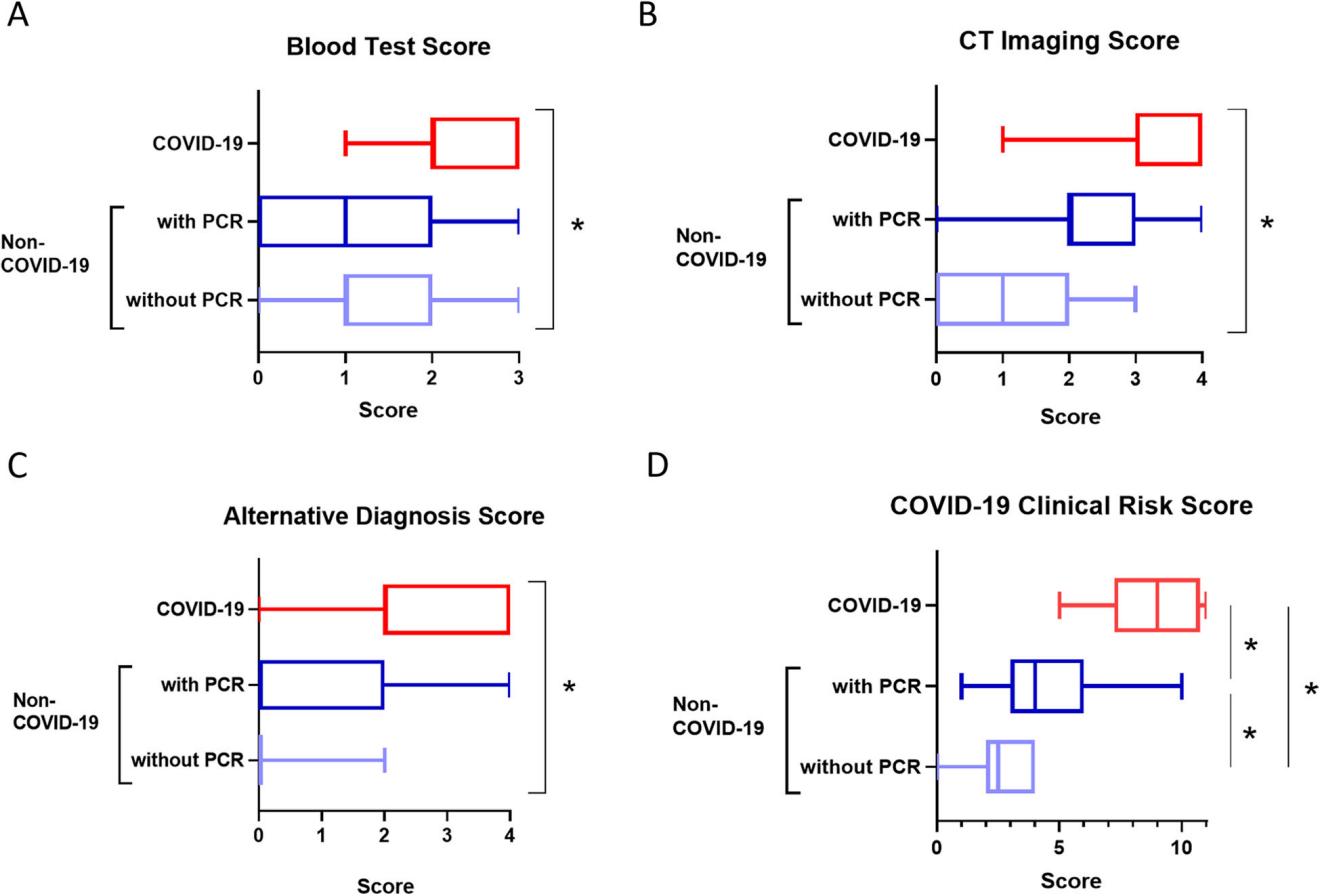
Distribution of each clinical score in patient groups. Bars, boxes, and lines represent Min to Max, interquartile range, and median, respectively. *P < 0.001

**Fig. 4:**
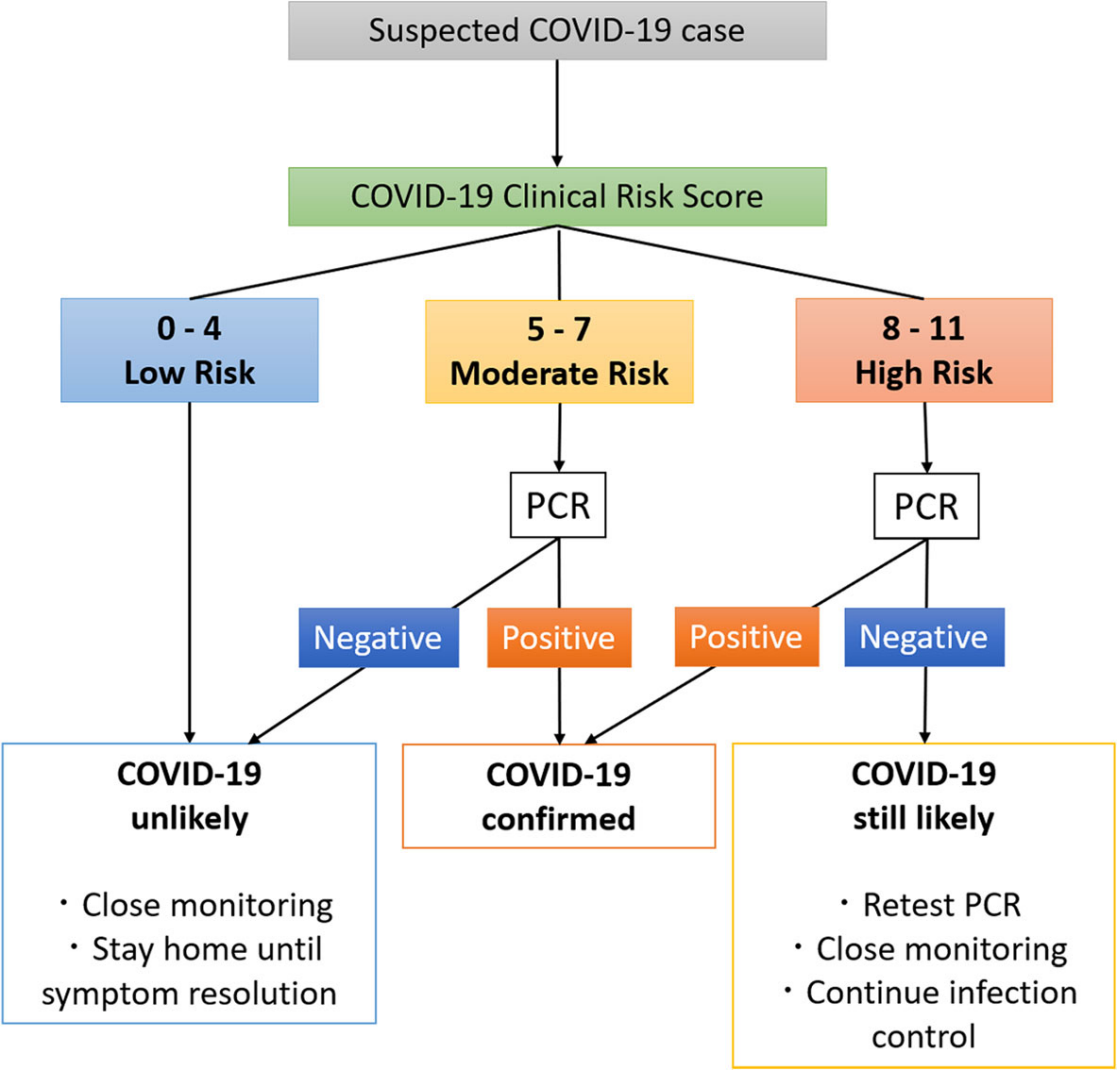
Patient risk classification based on “COVID-19 Clinical Risk Score” and a proposed practice algorithm

In light of the clinical differences between the COVID-19 and non-COVID-19 groups, we developed the “COVID-19 Clinical Risk Score” to determine whether the diagnosis of COVID-19 should be refuted or PCR testing should be carried out (Table 3). Based on the previous results, we decided to incorporate the following items into the clinical score: (A) blood test scores based on WBC count, eosinophil count, and procalcitonin value (maximum 3 points); (B) CT imaging scores (maximum 4 points); and (C) scores based on alternative diagnosis (maximum 4 points). Clinical scores were examined for three groups: non-COVID-19 patients who underwent PCR (non-COVID-19 with PCR group), those who did not undergo PCR (non-COVID-19 without PCR group), and confirmed COVID-19 patients. As shown in Fig. 3a-c, all blood test, CT imaging, and alternative diagnosis scores were higher in the COVID-19 group, but all clinical scores had overlapping distributions among the groups. We then summed all three scores and obtained the COVID-19 Clinical Risk Score (maximum 11 points). As a result, the distribution of the COVID-19 Clinical Risk Scores was clearly different among the groups (Fig. 3d). The non-COVID with PCR group had higher risk scores than the non-COVID without PCR group (mean 4.4 vs. 2.7, *P* < 0.001), which appeared to reflect the decision-making process in clinical practice. Furthermore, there was no overlap in the distribution of the risk scores between the non-COVID and COVID-19 groups (range, 0–4 vs. 5–11) (Fig. 3d). Finally, we propose a preliminary algorithm for the management of suspected COVID-19 patients based on the COVID-19 Clinical Risk Score (Fig. 4).

**Table 3:** COVID-19 Clinical Risk Score

COVID-19 Clinical Risk Score	Score
**Blood test score**	
WBC < 8000 (count/μL)	1
Eosinophil < 50 (count/μL)	1
Procalcitonin < 0.5 (ng/mL) and CRP ≥ 0.5 (mg/dL)	1
**CT imaging score**	
GGO with or without consolidation	1
Multilobar or bilateral lesions	1
Subpleural or lower lung dominant distribution	1
No atypical signs^a^	1
**Alternative diagnosis score (choose one)**	
More likely other diagnosis	0
Hard to determine	2
More likely COVID-19	4
**Total score**	Max 11

^a^atypical signs: consolidation without GGO, cavitation, nodules, tree-in-bud appearance, pleural effusion

## Discussion

In the present study, we analyzed the clinical characteristics of the medical management of suspected COVID-19 patients and compared the characteristics of suspected and confirmed COVID-19 patients who visited our medical institution. Patients who did not undergo PCR tended to have no history of high-risk behaviors, had lower CT scores, and had alternative diagnoses. There were significant differences in the COVID-19 group and the non-COVID-19 group with respect to some symptoms, blood test findings, CT score values, and the presence of alternative diagnoses. Based on our findings, we developed the COVID-19 Clinical Risk Score to guide proper management of suspected COVID-19 patients. As a result, the distribution of scores differed significantly among patient groups, with non-COVID-19 patients who did not undergo PCR scoring less than 4 and COVID-19 patients scoring greater than 5.

Although Hokkaido in Japan had been under the COVID-19 emergency alert during most of the study period, only two patients were confirmed to have COVID-19 among more than 100 suspected cases observed at our hospital. Because our facility is a university hospital with a wide range of specialized departments, the patient backgrounds and referral sources were diverse. Therefore, our patients’ backgrounds might be different from those of patients in primary care clinics and city hospitals. Nonetheless, COVID-19 is a disease with a high risk of outbreak and hospital shutdown once it is identified in a medical institution [22]. Even if the actual number of COVID-19 patients is low, there will be a large number of suspected cases. Thus, proper management of suspected COVID-19 cases is critical.

In this study, we compared the clinical characteristics of confirmed COVID-19 and non-COVID-19 patients. Significant differences between the two groups were found in the rates of sore throat and olfactory and gustatory disturbances. Although the symptoms of COVID-19 have been described as nonspecific, it has been recently found that olfactory and gustatory disturbances are characteristic of patients with COVID-19 [7, 23, 24]. The present study also confirmed that olfactory and taste disorders are more frequent in patients with COVID-19 than in suspected patients. However, the positivity rate was not high (13.3%); hence, these disorders were not included in the clinical risk score.

Leukopenia and lymphopenia have been reported as hallmarks of COVID-19 blood tests. The neutrophil-to-lymphocyte ratio tends to be higher in severe COVID-19, and lymphopenia is an important indicator of the severity of COVID-19 [25]. In the present study, COVID-19 patients had significantly lower WBC counts, and leukopenia was added to the risk score, but the rate of lymphocytopenia was not significantly different between COVID-19 and non-COVID-19 patients. Several non-COVID-19 patients had inflammatory diseases in which lymphocytes were depleted due to an elevated WBC count along with elevated neutrophil ratio; therefore, a low lymphocyte count may not be helpful in the diagnosis of COVID-19 in clinical practice. Contrary, eosinophil count has been reported to be decreased in the early stage of the disease and subsequently elevated in COVID-19 [26]. Several of the confirmed COVID-19 patients in this study also showed a significant decrease in eosinophil count, and eosinopenia was adapted in the risk score. Previous studies on COVID-19 have shown that elevated procalcitonin levels are associated with disease severity [10, 27]. However, in the present study, which compared COVID-19 cases with other febrile illnesses and cases with abnormal images on chest CT, a low level of procalcitonin was evident in patients with COVID-19, whereas the CRP level was elevated. Procalcitonin level is predominantly elevated in bacterial infections [28]. A substantial number of cases of bacterial infections may be observed in non-COVID-19 patients, and these patients were more likely to have elevated procalcitonin level at an early stage of the disease. Furthermore, we hypothesize that few cases of bacterial coinfection were observed in the COVID-19 patient group in the early stage of the disease. In the early stage of the disease, elevated procalcitonin appears to help reduce the risk of COVID-19. Thus, we selected leukopenia, eosinopenia, and low procalcitonin levels along with high CRP levels as useful routine blood tests for the diagnosis of COVID-19.

In the present study, we scored the chest CT findings and examined their usefulness in the diagnosis of COVID-19. Some of the CT findings characteristic of COVID-19, such as linear or rounded opacities, may be difficult for non-specialists to read [13–15]. Therefore, we focused on simple chest CT findings that could be read by general physicians and included the absence of atypical findings as an item of CT imaging score. As a result, there was a large difference in CT imaging scores between non-COVID-19 patients without PCR and confirmed COVID-19 patients. Even without professional CT reading, this result suggests that it is possible to distinguish COVID-19 from other diseases to some extent based on scoring chest CT findings.

Our focus in this study was to determine the risk of any disease besides COVID-19. This perspective is critical in the calculation of pretest probability prior to diagnostic testing, as exemplified by Well’s criteria used for the diagnosis of pulmonary embolism [29, 30]. COVID-19 is a disease with few specific clinical manifestations. Therefore, when only blood tests and CT findings are used as indicators for diagnosis, the score may underestimate or overestimate the likelihood of COVID-19. Based on the clinical course, patient background, and other clinical findings, we aimed to determine whether any other differential diagnoses would be more likely than COVID-19 and added this judgment to the COVID-19 Clinical Risk Score. The results showed a large difference in the risk scores between COVID-19 patients and non-COVID-19 patients. We believe that adding the judgment of clinicians regarding the differential diagnosis to objective indicators made the risk score clinically relevant.

The sensitivity of the PCR test is not sufficiently high with COVID-19, and if the PCR test alone is used as an indicator of judgment, several false-negative results will be missed. In addition, due to the limitations of medical and human resources, it is not always possible to perform PCR testing on all eligible patients. For appropriate medical care and infection control, it is reasonable to estimate the pretest probability based on the clinical score to determine whether PCR should be performed. The identification of patients who have a low risk of COVID-19 and are not eligible for PCR testing by the COVID-19 Clinical Risk Score is important for the proper use of the PCR test and to conserve resources. Patients with high scores should be carefully considered for retesting and continuing infection control, even if PCR results are negative. However, the actual decision to perform PCR will be influenced by a variety of factors, including the prevalence of COVID-19, severity of the patient’s illness, fear of medical staff or patients, and policies of medical institutions or administrative government. The clinical score should be used as a reference criterion, and decision-making should be flexible in the clinical setting. In addition, if sensitive and simple diagnostic tests are developed in the future, the implications of the clinical score will change. Such testing may take some time to develop, and for the time being, the COVID-19 Clinical Risk Score will be an important indicator in clinical practice.

This study had several limitations. First, since this was a retrospective study, a prospective validation is needed to prove the usefulness of the clinical risk score. Second, the backgrounds of the suspected and confirmed COVID-19 patients were different. Because only a low number of true COVID-19 patients were identified among the suspected cases, we used data from patients admitted for the treatment of COVID-19. Third, we cannot rule out the possibility that some of the patients we classified in the non-COVID-19 group might have had COVID-19. However, since only a single case of infection was identified in the subsequent clinical course of the patients without PCR testing and no nosocomial outbreaks occurred, we believe that our management was successful.

## Conclusion

We clarified the clinical features of patients with suspected COVID-19 treated at our institution and identified the clinical differences between suspected and confirmed COVID-19 patients. The COVID-19 Clinical Risk Score, based on blood test results, CT imaging findings, and the presence of an alternative diagnosis, was developed to demonstrate the validity of our practice. Larger sample size verification and prospective studies to examine the validity of clinical scores are required. We hope that the COVID-19 Clinical Risk Score will contribute to the improvement in the management of suspected COVID-19 cases in the future.

## Data Availability

The datasets used and/or analyzed during the current study are available from the corresponding author on reasonable request.

## Abbreviations

COVID-19: Coronavirus disease, 2019
CRP: C-reactive protein
CT: Computed tomography
GGO: Ground-glass opacity
IQR: Interquartile range
PCR: Polymerase chain reaction
SARS-CoV-2: Severe acute respiratory syndrome coronavirus 2
SD: Standard deviation
WBC: White blood cell
